# Efficacy of fecal microbiota transplantation in a patient with chronic intractable constipation

**DOI:** 10.1002/ccr3.1798

**Published:** 2018-09-09

**Authors:** Tadashi Ohara, Tatsuo Suzutani

**Affiliations:** ^1^ Department of Intestinal Bioscience and Medicine School of Medicine Fukushima Medical University Fukushima City Japan; ^2^ Department of Microbiology School of Medicine Fukushima Medical University Fukushima City Japan

**Keywords:** chronic intractable constipation, fecal microbiota transplantation, intestinal microbiota, short chain fatty acids (SCFAs), terminal fragment length polymorphism (T‐RFLP) method

## Abstract

We have presented the first case report of FMT therapy for a patient with chronic intractable constipation. This therapy resulted in good, medium‐term outcomes. Follow‐up analysis of the intestinal flora suggested that transplanted microbes from the donor, particularly *Bifidobacterium* and *Clostridium* cluster IX, may have been incorporated into the recipient.

## 1 INTRODUCTION

Fecal microbiota transplantation (FMT) therapy for patients with *Clostridium difficile* infection colitis has been found to have surprising efficacy.^1^ Dysbiosis of the intestinal flora is a problem and possible cause in diseases such as colorectal carcinoma, hepatocellular carcinoma, diabetes mellitus, obesity, and NASH.^2^, ^3^, ^4^, ^5^ In addition, dietary habits strongly affect the intestinal flora. Many patients have chronic constipation, and although these patients take laxatives and pre‐ and probiotics to improve their intestinal flora, the success of these approaches is limited. We used FMT therapy for a patient with chronic intractable constipation and achieved significant short‐ and medium‐term efficacy. This is the first case report of FMT therapy for such a patient.

## 2 CASE HISTORY/EXAMINATION

The patient was an 83‐year‐old male who had suffered with chronic intractable constipation for over fifty years. He had been treated with many anti‐constipation agents and probiotics, including magnesium oxide, carmellose sodium, D‐sorbitol, sodium picosulfate hydrate, and yogurt containing *Lactobacillus gasseri* 21, but the frequency of defecation remained at 7‐10 days and the stool volume was small, with a Bristle Stool Score (BSS) of 1 for classification of fecal properties. Based on an abdominal X‐ray, feces accumulated in the intestines, resulting in a very firm abdomen and anorexia. A general examination indicated that the patient had a mild Alzheimer's type of dementia that caused forgetfulness in daily life.

## 3 DIFFERENTIAL DIAGNOSIS/INVESTIGATION OF THERAPY

**Figure 1:**
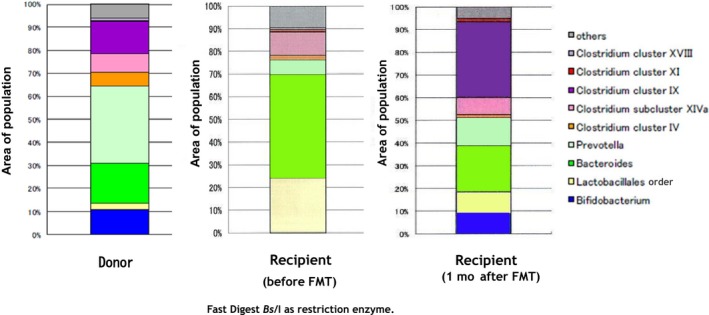
Each population area after treatment with Fast Digest *Bs*/I in donor and recipient microbiota

Colonoscopy findings did not show any diseases that could contribute to constipation, such as a tumor, polyp, or bowel stenosis. Mucosal melanosis was present, and was probably due to use of laxatives. We consulted with the patient and his family about treatment and reached a decision of FMT therapy. The intestinal flora of the patient and a 19‐year‐old healthy donor, who was the patient's nephew, were first examined (Figure 1 and Table 1). We confirmed the validity of the donor based on several infectious disease tests and the results of intestinal flora analysis. Also, the donor gave a guarantee to provide feces for transplant. The analysis of the patient's intestinal flora showed a depletion of both *Bifidobacterium* and some short chain fatty acids (SCFAs), especially acetic acid, propionic acid, and butyric acid (Table 2). The intestinal flora was analyzed by a terminal fragment length polymorphism (T‐RFLP) method after DNA extraction from feces.^6^, ^7^ The concentrations of seven SCFAs in the fecal samples were measured by gas chromatography‐mass spectrometry.^8^ The transplant‐microbial solution was prepared from the donor's feces,^1^ and then about 400 mL was infused into the recipient by colonoscopy. The solution was infused into the cecum to the ascending colon only once. FMT therapy was performed at Fukushima Daiichi Hospital.

**Table 1:** Classification groups analyzed by terminal fragment length polymorphism in donor and recipient microbiota

OUT	Classification group (%)	Donor	Recipient (before FMT)	Recipient (1 month after FMT)
106	*Clostridium* subcluster XIVa	0.0	0.0	0.0
110	*Clostridium* cluster IX	13.9	0.0	33.6
124	*Bifidobacterium*	10.8	0.0	8.8
137	*Prevotella*	0.0	0.0	0.0
168	*Clostridium* cluster IV	0.0	0.0	0.0
317	*Prevotella*	33.4	6.3	12.3
332	*Lactobacillus* order	1.2	1.4	2.5
338	*Clostridium* cluster	0.5	1.1	1.4
366	*Bacteroides*	9.9	6.3	6.2
369	*Clostridium* cluster IV	0.0	1.7	0.0
423	*Clostridium* cluster X VIII	0.0	0.0	0.0
443	None	0.0	0.6	0.0
469	*Bacteroides*	5.7	37.6	14.2
494	*Clostridium* subcluster XIVa	1.8	3.6	3.8
505	*Clostridium* subcluster XIVa	0.0	0.0	0.0
517	*Clostridium* subcluster XIVa	0.0	0.0	0.0
520	*Lactobacillus* order	0.0	5.0	0.0
641	None	0.0	0.0	0.0
650	*Clostridium* cluster XVIII	0.9	0.9	0.0
657	*Lactobacillu*s order	1.6	17.9	7.1
749	*Clostridium* cluster IV	6.1	0.4	1.4
754	*Clostridium* subcluster XIVa	1.2	1.8	0.6
770	None	0.9	0.7	0.0
853	*Bacteroides*	1.8	1.6	0.0
919	*Clostridium* cluster IX	2.2	4.6	3.6
940	*Clostridium* subcluster	2.6	2.7	1.4
955	*Clostridium* subcluster XIVa	1.8	1.0	0.0
968	None	0.5	0.8	0.0
990	*Clostridium* subcluster XIVa	3.2	3.8	3.1

OUT indicates operational taxonomic unit.

**Table 2:** Production of short chain fatty acids

SCFA	Donor	Recipient (before FMT)
Acetic acid	57.2	31.5
Propionic acid	23.3	4.9
Butyric acid	14.4	4.4
Isobutyric acid	0.6	1.6
Valeric acid	2.3	1.0
Isovaleric acid	0.7	2.0
Caproic acid	1.0	

Data are shown as concentrations (μmol/g). A blank indicates a value lower than the limit of detection (LOD). The LODs for acetic acid, propionic acid and butyric acid were 2.0, 0.7 and 0.7 μmol/g, respectively. The LODs for isobutyric acid, valeric acid, isovaleric acid and caproic acid were all 0.3 μmol/g.

## 4 OUTCOME AND FOLLOW‐UP

Immediately after FMT therapy, the recipient defecated every day and developed abdominal distensions without the need for drug therapy. The fecal properties and bowel movements are summarized in Table 3. The efficacy of the FMT therapy was remarkable and continued for 1 month. An examination of the patient's intestinal flora at 1 month after FMT therapy showed that the composition resembled that of the donor, with a notable increase in the populations of *Bifidobacterium* and *Clostridium* cluster IX in the recipient (Figure 1 and Table 1). The patient's dementia symptoms of forgetfulness also showed a minor improvement after FMT therapy. The positive effects of FMT therapy on normal bowel movements, frequent passage, and normal fecal properties were still present after more than 11 months.

**Table 3:** Changes of fecal properties and bowel movements in the recipient before and after FMT therapy

Item	Before FMT	After FMT
Abdominal distension	Marked	None
Borborygmus feeling	Almost nothing	Almost normal
Use of laxative	Many laxatives	None
Feces frequency	0‐1/week	1/day
Feces weight	Minimum	Moderate
Feces odor	Offensive	Mild
Feces color	Blackish‐brown	Yellowish‐brown
Bristol Stool Scale	1	3‐4

## 5 DISCUSSION

Bowel movements are accelerated by SCFAs such as butyric acid and propionic acid,^8^ and SCFAs^9^ are generated by *Bifidobacterium*,* Lactobacillus*, various types of *clostridium* clusters, and intestinal flora in general. Incorporation of transplanted microbes after FMT therapy has not been reported, but the results from our follow‐up examination suggested that *Bifidobacterium* and *Clostridium* cluster IX were incorporated into the recipient's intestinal flora. This beneficial effect of FMT therapy may be applicable for other diseases, such as diabetes mellitus, inflammatory bowel disease, and dementia. We plan to perform a full analysis of the incorporated microbes in a further study.

## 6 CONCLUSIONS

We have presented the first case report of FMT therapy for a patient with chronic intractable constipation. This therapy resulted in good short‐ and medium‐term outcomes. Follow‐up analysis of the intestinal flora suggested that transplanted microbes from the donor, particularly *Bifidobacterium* and *Clostridium* cluster IX, may have been incorporated into the recipient's intestinal flora analyzed by T‐RFLP method. It may be a possibility that the further follow‐up observation and the detailed full analysis of microbes in this case lead to the new developments of FMT treatment.

## CONFLICT OF INTEREST

The authors declare that they have no other competing interests.

## AUTHOR CONTRIBUTIONS

Tadashi Ohara, M.D., Ph.D.is a researcher of gastrointestinal pathology, and Professor of Department of Intestinal Bioscience and Medicine, Fukushima Medical University. Tatsuo Suzutani, M.D., Ph.D.is a researcher of microbiology, and Professor of Department of Microbiology and Department of Intestinal Bioscience and Medicine, Fukushima Medical University. Authors read and approved the final manuscript.
